# Concurrent light chain and transthyretin cardiac amyloidosis: A case report and review of the literature

**DOI:** 10.1097/MD.0000000000046900

**Published:** 2026-05-12

**Authors:** Huanxin Sun, Ning Zhang, Xin Liu, Miao Wang, Liren Wang, Yonghong Li

**Affiliations:** a Department of Cardiology, the Affiliated Hospital of Qingdao University, Qingdao, Shandong, China.

**Keywords:** cardiac amyloidosis, concurrent, endomyocardial biopsy, light chain, transthyretin

## Abstract

**Rationale:**

Cardiac amyloidosis is caused by extracellular deposition of amyloid proteins, with over 30 distinct forms identified based on protein composition. The predominant types are immunoglobulin light chain and transthyretin (ATTR) amyloidosis. This study presents an exceedingly rare case of concurrent light chain cardiac amyloidosis (AL-CA) and ATTR cardiac amyloidosis.

**Patient concerns:**

A 69-year-old Chinese woman was admitted to the hospital due to recurrent heart failure that had been present for the past 3 months. She presented with symptoms including fatigue, reduced exercise tolerance, and difficulty breathing when reclining flat. She exhibited inadequate response to anti-heart failure therapy.

**Diagnoses:**

The diagnosis of coexisting AL-CA and wild-type transthyretin cardiac amyloidosis was confirmed through a combination of echocardiography, blood tests, cardiovascular magnetic resonance imaging, technetium-99m pyrophosphate scintigraphy, invasive procedures (bone marrow and endomyocardial biopsies), mass spectrometry, and genetic testing.

**Interventions:**

Prior to diagnosis, the patient was treated for heart failure with preserved ejection fraction with diuretics, sodium-glucose cotransporter-2 inhibitors, and angiotensin receptor-neprilysin inhibitors. Following confirmation of cardiac amyloidosis, tafamidis was initiated to stabilize ATTR. Chemotherapy was subsequently attempted after hematologic referral but discontinued due to adverse effects. The patient later ceased most prescribed therapies in favor of unproven alternatives.

**Outcomes:**

After 3 months of comprehensive treatment, the patient discontinued tafamidis and other related therapies due to intolerance of adverse effects, opting instead for traditional Chinese medicine. Subsequent poor treatment adherence prevented regular follow-up and reassessment. Although the patient reported persistent exertional dyspnea during sporadic follow-up visits, no objective disease progression parameters (e.g., N-terminal pro-B-type natriuretic peptide levels, cardiac imaging changes) or standardized treatment response data were obtained.

**Lessons:**

We report a confirmed case of coexistent AL-CA and wild-type transthyretin cardiac amyloidosis, validated through comprehensive diagnostic evaluation. Although dual therapy targeting both amyloid types may be needed, the lack of guidelines and follow-up data highlights the need for standardized management approaches. Future studies should prioritize long-term outcome tracking.

## 1. Introduction

Cardiac amyloidosis (CA) is caused by the deposition of amyloid proteins outside myocardial cells. Over 30 types of abnormal protein deposits can lead to CA according to the protein composition. CA can be classified into types such as immunoglobulin light chain (AL) and transthyretin (ATTR).^[1]^ The majority of CA cases are of the AL or ATTR type. The coexistence of both AL and ATTR types is extremely rare. Here we report a case of CA with a coexistence of AL and ATTR types.

## 2. Case presentation

A 69-year-old Chinese woman presented with a 3-month history of progressive dyspnea and chest tightness, with symptomatic deterioration to New York Heart Association Class II over the past month. She reported new-onset orthopnea, significantly reduced exercise tolerance and persistent fatigue. Her medical history was notable for myocarditis at age 30 without subsequent cardiac complications. Despite receiving symptomatic treatment at a local hospital (details unavailable), her symptoms persisted and eventually necessitated hospitalization.

**Figure 1.**
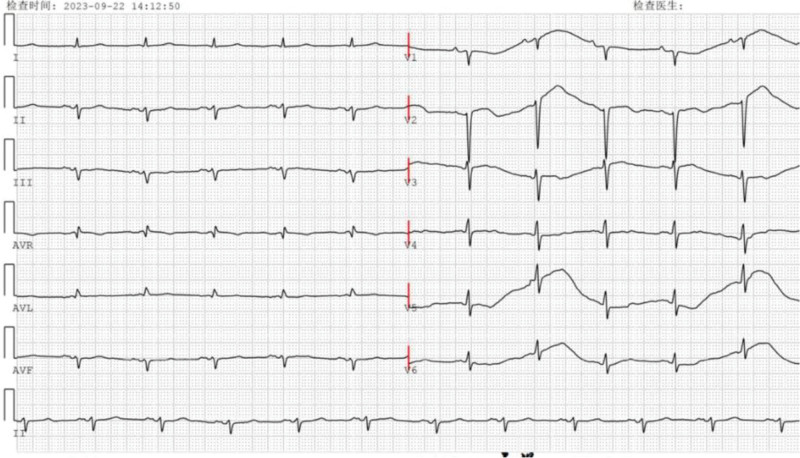
Electrocardiogram showed left axis deviation and low voltage QRS.

On admission, vital signs were stable (blood pressure 120/64 mm Hg, heart rate 86 bpm) with bilateral basilar rales extending to the mid-lung fields. Electrocardiogram revealed sinus rhythm with left axis deviation, low limb lead voltage and diffuse nonspecific ST-T wave abnormalities (Fig. 1). Laboratory investigations revealed elevated cardiac biomarkers including troponin I and N-terminal pro-B-type natriuretic peptide (NT-pro BNP; Table 1). Other parameters including renal function, hemoglobin levels, and serum calcium remained within normal ranges.

**Table 1:** Serial measurements of NT-pro BNP and cardiac troponin I levels.

Laboratory results	Oct 10th, 2023	Oct 14th, 2023	Dec 10th, 2023
NT-pro BNP (pg/mL)	5044.00	4511.00	2715
Cardiac troponin I (ng/mL)	0.034	–	<0.017

“–” = data not available at that time, NT-pro BNP = N-terminal pro-B-type natriuretic peptide.

**Figure 2.**
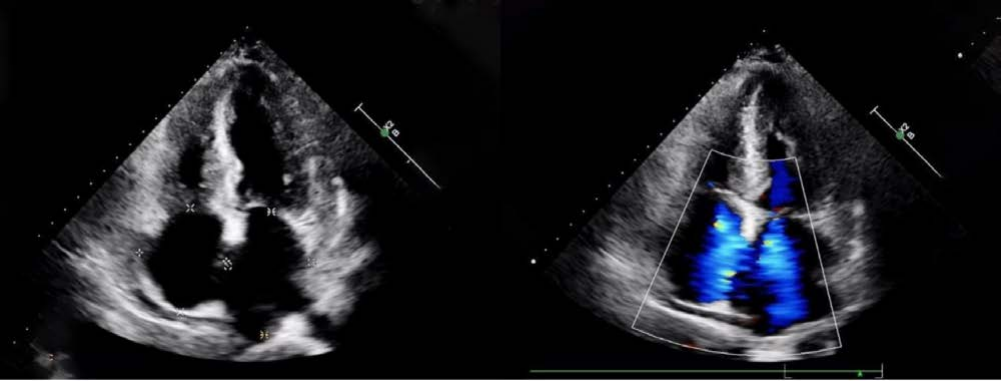
Echocardiography showed bilateral atrial enlargement.

**Figure 3.**
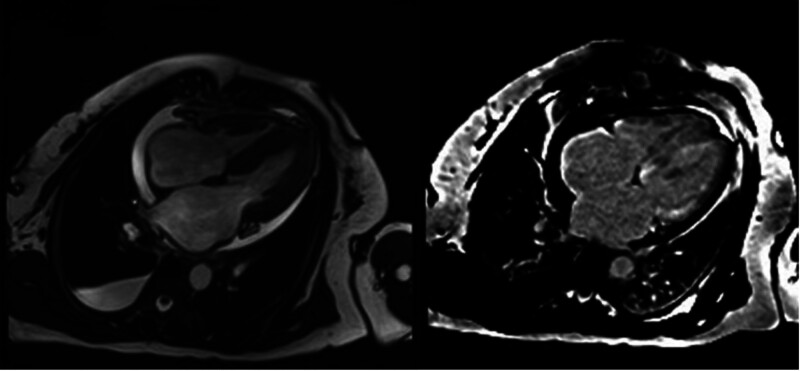
CMR showed bilateral atrial enlargement and late gadolinium enhancement. CMR = cardiovascular magnetic resonance.

Transthoracic echocardiography revealed a preserved left ventricular ejection fraction (65%), bilateral atrial enlargement, and symmetric thickening of the interventricular septum and left ventricular free wall. The myocardium exhibited granular sparkling echogenicity, a characteristic finding suggestive of CA (Fig. 2).^[2]^ In light of the patient’s heart failure symptoms, elevated cardiac biomarkers, electrocardiographic abnormalities, and echocardiographic features, CA was suspected as the underlying cause. To further evaluate myocardial infiltration, cardiovascular magnetic resonance (CMR) imaging was performed. It showed biatrial enlargement and subendocardial diffuse late gadolinium enhancement, with additional enhancement of the right atrial wall (Fig. 3), supporting the initial suspicion of CA.^[3]^

At this point, the probability of CA was considered high. However, given the divergent treatment strategies for different amyloid subtypes – particularly AL and ATTR – further investigations were warranted. The patient underwent a comprehensive hematologic workup and technetium-99m pyrophosphate (99mTc-PYP) scintigraphy.

The hematologic tests included serum and urine protein electrophoresis with immunofixation and quantification of serum free light chains (FLCs). Although the urine immunofixation electrophoresis was negative, serum immunofixation and FLCs assays revealed distinct abnormalities (Table 2), indicating the presence of a monoclonal AL and raising strong suspicion of light chain cardiac amyloidosis (AL-CA).^[4]^ To further characterize the clonal plasma cell proliferation, a bone marrow aspiration was performed, which identified 14% plasma cells. Flow cytometric analysis detected an abnormal plasma cell population accounting for 1.45% of total cells, exhibiting CD38++/CD138++ positivity and cytoplasmic kappa light chain restriction. These collective findings support a diagnosis of light chain multiple myeloma.^[5]^

**Table 2:** Patient’s longitudinal hematology data.

Laboratory results	Sep 4th, 2023	Sep 23rd, 2023
M protein (g/L)	3.5	2.4
κ-FLC (mg/L)	760	710
λ-FLC (mg/L)	1990	1610
rFLC (κ-FLC/λ-FLC)	2.62	2.27
dFLC ( κ-FLC-λ-FLC ) (mg/L)	1230	900

FLC = free light chain.

**Figure 4.**
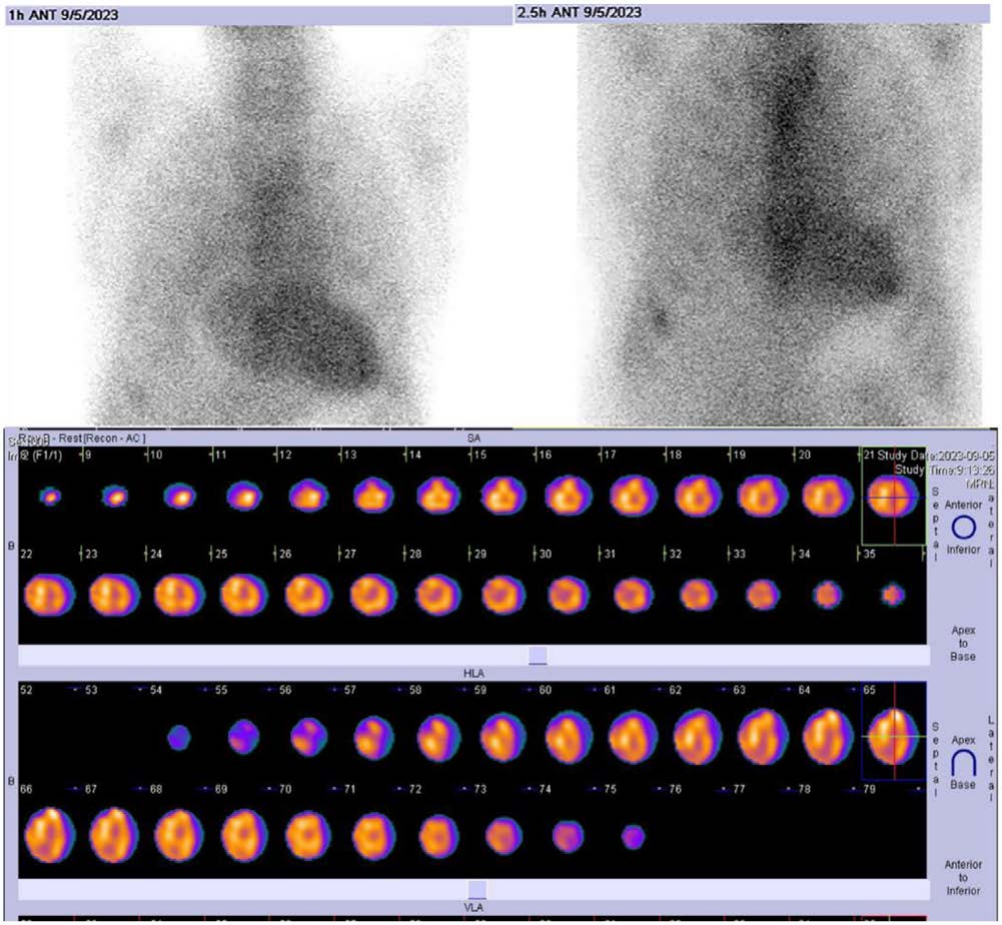
99mTc-PYP shows grade 3 cardiac uptake. 99mTc-PYP = technetium-99m pyrophosphate.

Notably, 99mTc-PYP scintigraphy showed findings suggestive of transthyretin cardiac amyloidosis (ATTR-CA). At 3 hours postinjection, the heart-to-contralateral lung uptake ratio was 1.37, with Perugini Grade 3 myocardial uptake exceeding rib uptake (Fig. 4). While such findings typically confirm ATTR-CA in the absence of monoclonal protein, the conflicting hematologic profile necessitated endomyocardial biopsy for definitive diagnosis.^[6–8]^

**Figure 5.**
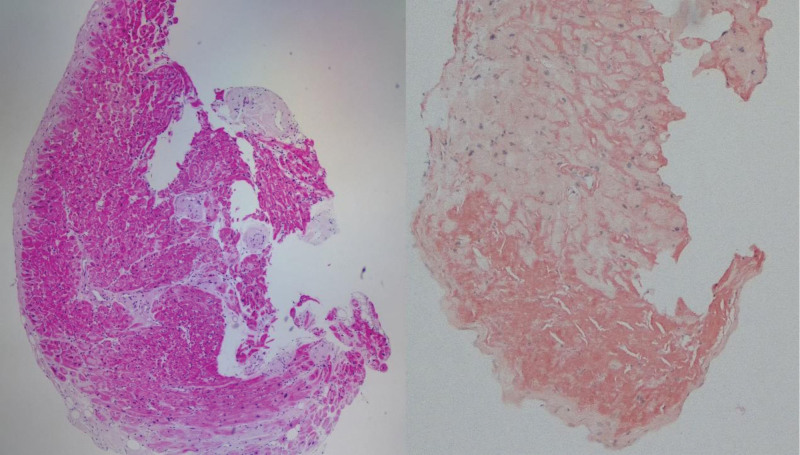
The left side shows the HE staining image of myocardial tissue, and the right side shows the Congo red staining image of myocardial tissue.

**Figure 6.**
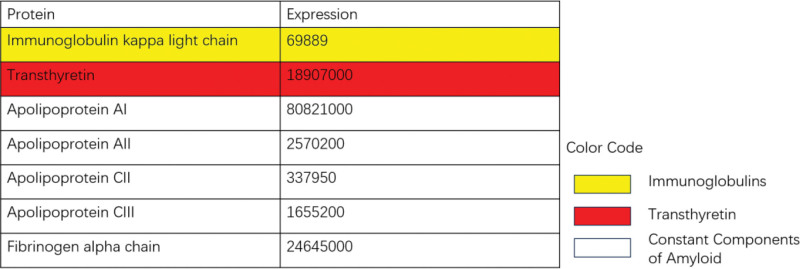
Results of protein mass spectrometry analysis. Apolipoprotein A-I (AApoAI), Apolipoprotein A- II (AApoAII), ApoC- II (Apolipoprotein CII), ApoC -III (Apolipoprotein C III), and FGN (Fibrinogen alpha chain) are visible (white) in cases of CA. Additionally, in this patient’s sample, Transthyretin (red) and immunoglobulin light chains (yellow) are also visible.

Comprehensive diagnostic evaluation supported the coexistence of AL-CA and ATTR-CA. Although 99mTc-PYP scintigraphy is highly sensitive and specific for ATTR-CA, histological confirmation is essential due to the potential for false positives.^[9–11]^ Endomyocardial biopsy confirmed myocardial amyloid deposition (Fig. 5),^[12]^ and mass spectrometry identified both transthyretin and ALs (Fig. 6). Genetic testing revealed no pathogenic variants. These findings confirmed a rare case of coexisting wild-type transthyretin cardiac amyloidosis (ATTRwt-CA) and AL-CA.^[13]^

## 3. Treatment and management

The patient received comprehensive therapy addressing 3 key components of CA management: Directed diuretic therapy for heart failure (furosemide 20 mg bid, later oral; spironolactone 20 mg bid) and sodium-glucose cotransporter-2 inhibitors (dapagliflozin 5 mg daily), while Angiotensin receptor-neprilysin inhibitors (sacubitril/valsartan 25 mg bid) and beta-blockers (metoprolol succinate 23.75 mg daily) were cautiously titrated and discontinued due to hypotension; arrhythmia control with edoxaban 30 mg daily and amiodarone 200 mg daily for paroxysmal atrial fibrillation, plus reintroduced low-dose metoprolol for rate control; and etiology-specific therapy including tafamidis 61 mg daily for ATTR stabilization and daratumumab/dexamethasone for AL-CA (aborted due to toxicity).

After taking 3 months of tafamidis, the patient stopped most medications and chose traditional Chinese medicine (components unknown) and intermittent metoprolol. Because of her poor adherence, the key prognostic markers like NT-pro BNP and serum FLCs were only those during hospitalization at our center (see Tables 1 and 2). The patient reject any monitoring.

## 4. Discussion

CA is a restrictive cardiomyopathy caused by the deposition of abnormal proteins within the myocardium.^[1]^ Due to its nonspecific clinical manifestations, CA is frequently underdiagnosed. Most confirmed cases present as heart failure with preserved ejection fraction of unknown etiology.^[12,14]^

Based on our diagnostic experience with this patient, we summarized the diagnostic algorithm for CA. Patients presenting with unexplained heart failure require systematic evaluation for CA, beginning with electrocardiogram, echocardiography, and cardiac biomarkers (NT-pro BNP/troponin). If these initial tests raise suspicion, further hematological tests – including serum FLCs, protein electrophoresis, and immunofixation – as well as 99mTc-PYP scintigraphy should be performed. Depending on the comprehensive results, endomyocardial or bone marrow biopsy may be considered to establish a definitive diagnosis.

Speckle-tracking echocardiography is a crucial noninvasive imaging tool in the diagnosis of CA.^[15]^ It is particularly valuable for detecting significant impairment in global longitudinal strain at early stages, even when left ventricular ejection fraction is preserved, indicating subclinical myocardial dysfunction.^[16]^ Its most characteristic finding is the “apical-sparing” pattern, where longitudinal strain is severely reduced in the basal and mid-ventricular segments but relatively preserved in the apical segment. This feature aids in differentiating CA from other forms of myocardial hypertrophy, such as hypertensive heart disease or hypertrophic cardiomyopathy. Therefore, speckle-tracking echocardiography should be employed as a first-line screening tool in suspected CA cases.^[17]^

Critical considerations in 99mTc-PYP scintigraphy interpretation include its high sensitivity (>99%) and specificity (82–86%) for ATTR-CA, with studies demonstrating Grade 2 to 3 uptake in up to 22% of AL-CA cases (false positives).^[18,19]^ Potential causes of false-positive 99mTc-PYP scintigraphy in the diagnosis of ATTR-CA include recent myocardial infarction (<4 weeks), rib fractures, valvular or annular calcification, AL-CA, apolipoprotein-related amyloidosis (Apolipoprotein A-I, Apolipoprotein A-II, Apolipoprotein A-IV, and β2‐Microglobulin Amyloidosis), hypertrophic cardiomyopathy, hydroxychloroquine-induced cardiotoxicity, and cardiac blood pool activity.^[11,18,20]^ In this patient, if 99mTc-PYP scintigraphy was falsely positive, coexisting AL-CA would be the most likely explanation, as other causes were effectively excluded. Therefore, endomyocardial biopsy was essential. In general, all 99mTc-PYP scintigraphy results require thorough exclusion of AL-CA, ideally through multimodality imaging (echocardiography, CMR, and SPECT-CT).^[21]^ Biopsy remains critical in diagnostically challenging cases.^[21]^

Following confirmation of amyloid deposition, precise identification of the precursor protein is essential.^[21]^ While immunohistochemistry provides initial protein characterization, its reliability is limited in CA.^[22]^ For definitive subtyping, laser microdissection with tandem mass spectrometry offers superior accuracy in distinguishing amyloid fibril proteins from associated components, serving as the gold standard for diagnostically challenging cases.^[21]^ Additionally, all patients diagnosed with ATTR-CA should undergo transthyretin genetic testing to distinguish wild-type (ATTRwt) from mutant ATTR forms.^[11]^

Accurate subtyping of CA is essential for guiding individualized therapeutic strategies.^[13,23]^ In this case, we successfully diagnosed a rare co-occurrence of AL and ATTRwt amyloidosis through a systematic multimodal diagnostic approach, including serum immunofixation electrophoresis, serum FLCs, 99mTc-PYP scintigraphy, and laser microdissection with tandem mass spectrometry of endomyocardial biopsy specimens.

It must be emphasized that although a treatment plan was formulated based on the diagnostic findings, the patient’s poor adherence resulted in a lack of serial post-therapeutic cardiac biomarkers (including NT-pro BNP and high-sensitivity troponin) and imaging follow-up data. This limitation precludes any objective assessment of treatment efficacy. Nevertheless, this case holds significant diagnostic value: First, it reinforces the critical role of a multimodal diagnostic strategy in differentiating complex amyloid subtypes. Second, it highlights the unique advantages of a multidisciplinary team approach in optimizing clinical decision-making for such challenging cases.^[24]^

Although the coexistence of AL-CA and ATTR-CA is exceedingly rare, Gami et al reported 3 such cases in a 2024 case series.^[25]^ The present case shares several key features with those described by Gami, including unexplained heart failure accompanied by typical “red flag” manifestations such as low electrocardiographic voltage, atrial fibrillation, and biatrial enlargement, with all diagnoses confirmed by endomyocardial biopsy.^[26]^ However, notable differences exist: the patient is a 69-year-old Asian woman, significantly younger than those in the Gami cohort (aged 83–90 years). Furthermore, she lacked systemic involvement such as carpal tunnel syndrome or lumbar spinal stenosis. Particularly noteworthy is her history of myocarditis, which we hypothesize may have served as a predisposing factor contributing to the relatively early onset of ATTRwt, although a direct causal relationship between remote myocardial injury and ATTRwt pathogenesis has not been definitively established in the literature. Consistent with the findings of Gami et al, this case underscores the diagnostic challenges posed by overlapping amyloid subtypes and highlights the complexity of disease coexistence. We anticipate that this case, together with previously published reports, will enhance clinical recognition of potential AL and ATTR co-amyloidosis, promote vigilance toward “red flag” signs, and thereby help reduce underdiagnosis, facilitating earlier and more accurate diagnosis.

## 5. Conclusion

This case highlights the rare coexistence of AL-CA and ATTRwt-CA, a combination scarcely reported in the literature. The diagnostic complexity of such dual pathology underscores the necessity for a comprehensive and systematic approach, including multimodal imaging, endomyocardial biopsy, proteomic analysis, and genetic testing. Clinicians should maintain a high index of suspicion when clinical, imaging, or laboratory features are discordant. Early and accurate identification of concurrent amyloid subtypes is essential, as it directly impacts treatment strategy and prognosis.

## 6. Limitation

This study has 3 principal limitations that warrant consideration. First, the complete absence of longitudinal biomarker (e.g., serial NT-pro BNP and troponin) and imaging (e.g., echocardiographic and CMR) data following diagnosis prevents any objective evaluation of disease progression or treatment response. Second, as a single-center case report, the generalizability of our findings remains constrained by the unique characteristics of this individual presentation. Third, while the diagnostic pathway for dual amyloidosis can be comprehensively described, the lack of outcome data significantly restricts our ability to derive clinically actionable management recommendations.

## Acknowledgments

We thank the patient for allowing the publication of this case report.

## Author contributions

**Investigation**: Ning Zhang, Xin Liu, Yonghong Li.

**Methodology**: Xin Liu.

**Resources**: Xin Liu, Yonghong Li.

**Visualization**: Ning Zhang, Xin Liu.

**Writing – original draft**: Huanxin Sun, Miao Wang, Liren Wang.

**Writing – review & editing**: Yonghong Li.
